# A Value Chain Approach to Characterize the Chicken Sub-sector in Pakistan

**DOI:** 10.3389/fvets.2020.00361

**Published:** 2020-07-03

**Authors:** Hassaan Bin Aslam, Pablo Alarcon, Tahir Yaqub, Munir Iqbal, Barbara Häsler

**Affiliations:** ^1^Veterinary Epidemiology, Economics and Public Health Group, Department of Pathobiology and Population Sciences, Royal Veterinary College (RVC), London, United Kingdom; ^2^Avian Influenza Virus Group, The Pirbright Institute, Woking, United Kingdom; ^3^Department of Microbiology, University of Veterinary and Animal Sciences, Lahore, Pakistan

**Keywords:** Pakistan, chickens, production systems, mapping, value chains

## Abstract

The chicken industry of Pakistan is a major livestock sub-sector, playing a pivotal role in economic growth and rural development. This study aimed to characterize and map the structure of broiler and layer production systems, associated value chains, and chicken disease management in Pakistan. Qualitative data were collected in 23 key informant interviews and one focus group discussion on the types of production systems, inputs, outputs, value addition, market dynamics, and disease management. Quantitative data on proportions of commodity flows were also obtained. Value chain maps were generated to illustrate stakeholder groups and their linkages, as well as flows of birds and products. Thematic analysis was conducted to explain the functionality of the processes, governance, and disease management. Major chicken production systems were: (1) Environmentally controlled production (97–98%) and (2) Open-sided house production (2–3%). Broiler management systems were classified as (I) Independent broiler production; (II) Partially integrated broiler production; and (III) Fully integrated broiler production, accounting for 65–75, 15–20, and 10–15% of commercial broiler meat supply, respectively. The management systems for layers were classified as (I) Partially integrated layer production and (II) Independent layer production, accounting for 10 and 80–85% in the egg production, respectively. The share of backyard birds for meat and eggs was 10–15%. Independent, and integrated systems for chicken production could be categorized in terms of value chain management, dominance of actors, type of finished product and target customers involved. Integrated systems predominantly targeted high-income customers and used formal infrastructure. Numerous informal chains were identified in independent and some partially integrated systems, with middlemen playing a key role in the distribution of finished birds and eggs. Structural deficiencies in terms of poor farm management, lack of regulations for ensuring good farming practices and price fixing of products were key themes identified. Both private and public stakeholders were found to have essential roles in passive disease surveillance, strategy development and provision of health consultancies. This study provides a foundation for policy-makers and stakeholders to investigate disease transmission, its impact and control and the structural deficiencies identified could inform interventions to improve performance of the poultry sector in Pakistan.

## Introduction

Chicken production is an important sub-sector of agriculture in Pakistan and plays a pivotal role in rural economic development. The estimated number of commercial chickens in the country in 2017 was 1,022 million birds with production of 17,083 million eggs and 1,270,000 tons of meat, providing direct and indirect employment to over 1.5 million people (1, 2). The commercial chicken industry in Pakistan has grown at 8–10% annually over the past two decades (3). The efficiency and availability of modern farming technologies, high profit margins, and the establishment of federal institutions for poultry production in the 1990s were important drivers for the modern chicken industry in Pakistan (4). The growth of the livestock industry in low and middle income countries is determined by a rise in the total number of livestock, whereas per animal carcass weight is the key indicator in high income countries (5). In 2015, Pakistan was the 11th largest chicken producer in the world on the basis of number of birds produced (2, 6). Since then, investment by private and public sectors has increased, from 200 billion Pakistani rupee (PKR) (1.28 billion USD) in 2015 to 700 billion PKR (4.47 billion USD) in 2018.

The initial rise (1960–1980) in growth of the chicken industry was promising but not sustained. Outbreaks of infectious diseases like hydropericardium syndrome, infectious bursal disease (4) and avian influenza (AI) (7) caused important production losses and trade embargos (8). This triggered a shift in the chicken production sector toward more industrial production with farmers relocating their poultry production units into cooler and more bio-secured hilly northern areas of Pakistan and switching to environmentally controlled houses (4).

Chicken meat accounts for 32.7% of the total meat production in Pakistan (2), 70% of which is produced in the Punjab province (3). Consumption of chicken meat is growing steadily in Pakistan because of its low price (beef is over 20% and mutton is over 50% more expensive) and low fat content (3, 9). It is also attractive to value chain actors because of a short production cycle and easier processing of carcasses due to being smaller size than alternative meats such as mutton and beef (3, 6). Despite this growth, the average chicken product consumption per capita in Pakistan, a low and middle income country, is 5 kilograms of meat and 51 eggs per annum, whereas in high income countries it is 40 kilograms of meat and 300 eggs annually (8). The current standing population of 1,560 million broilers and 60 million layers is still insufficient to meet local needs for meat and eggs (9). As more people are consuming halal (the prescribed method of slaughter under Islamic law) meat globally, there is also an opportunity for Pakistan to increase its halal chicken meat export across the world.

The poultry industry in Pakistan is constantly evolving supported by government in form of tax reliefs, passing of the Punjab Poultry Production Act (10) and development of appropriate slaughter houses (6). Its growth has offered opportunities for national and international investors. The rapidly growing population, along with the influx of people to urban areas, and changes in people's eating habits, are creating business opportunities for animal protein producers in Pakistan (11). Increases in the domestic price of red meat, due to its fluctuating local and export markets, drastic changes in local supply and demand, and economic instability of the country (12) are further driving developments in the chicken production sector. Increasing investments in the chicken production industry, along with the expansion of chicken sales networks, are responsible for the reduced prices of chicken and its products, making chicken meat and eggs some of the cheapest and most consumed sources of animal protein in the country (8).

There is a dearth of modern value chain tactics in the supply and marketing of chicken and its products (6) for most of the poultry produced in the country. Despite increased production, there is limited vertical integration. Structural inefficiencies in terms of fragmented broiler and layer production and weak institutional environments result in a lack of coordination in terms of production, pricing, and marketing decisions for chicken meat and eggs (13). These discrepancies are further potentiated by the lack of scientific, hygienic methods to process poultry meat and eggs at the retail level, and a scarcity of capacity in poultry meat bioscience and technology which could hinder future development of the industry (3).

Mapping of production systems can provide an overview and understanding of the various production, harvesting, and distribution steps, types of actors and products involved along with their hierarchal position in value chains (14). Furthermore, the analysis of livestock value chains develops understanding of the operations, structural inefficiencies and identification of critical points for potential policy interventions (15). Despite the growing poultry industry and its importance in providing affordable and healthy protein in form of meat and eggs, no study has yet mapped poultry value chains in Pakistan.

Chaudhry et al. (13) studied pricing mechanisms in commercial broiler value chains and found the industry at the brink of crisis due to strong price fluctuations. Jalil et al. (12) also studied meat value chains in smallholders in Pakistan and found large transportation costs that were responsible for high prices of red and white meat. Hence, there is a need to investigate the detailed structure of the broiler and layer value chains in order to understand and target the intervention points for disease and value chain management that can support economic resilience and food safety within these chains. This is of particular importance due to the presence of continuous fluctuations in the price of inputs for chicken farming and of infectious diseases like AI threatening the efficiency and safety of the system. Value chain studies have been recommended in the development of strategies to prevent and control AI, especially in East Asian countries (16, 17) and to measure disease and intervention effects in these systems.

The main aims of this study were to characterize and map the commercial broiler and layer production systems and the value chains associated with these systems and to investigate options for chicken disease management and reporting in different production systems in Pakistan. The outcomes of this study generate information relevant for stakeholders, directly and indirectly involved in chicken production, who could be interested in identifying ways to improve value chain operations and design efficient disease control strategies.

## Materials and Methods

Broiler, layer, and backyard chicken production systems and their value chains were studied by collecting qualitative and quantitative data between October 2017 and December 2017 mainly by key informant interviews (KIIs) and one focus group discussion (FGD). Activities included (a) identifying the various value chain systems; (b) investigating their contribution to the total meat and egg production in the country; (c) mapping and describing the meat and egg value chains of different broiler and layer production systems. The latter included characterizing types of stakeholders, products and flows in the value chains, and identifying the services and measures taken for diseases like AI prevention and control.

### Study Area and Selection of Participants

Punjab province was selected as the study area, because it accounts for the highest share in broiler and layer production in Pakistan with 608 million (63.25%) and 28.46 million (58.20%) out of 961.5 and 48.83 million broiler and layer birds, respectively (1). The province is the base for major poultry companies in the country and therefore represents the ideal site to access key informants of different production systems.

The major chicken producing areas of central and north Punjab and a list of stakeholders to be interviewed were identified during informal discussion with poultry experts at the University of Veterinary and Animal Sciences Lahore and Poultry Research Institute in Rawalpindi, Pakistan. Target interviewees included federal and provincial poultry research officials, commercial chicken producing farmers, backyard farmers, poultry, and egg traders and owners of vertically integrated and processing companies (Table 1). They were selected such to have a broad representation from all parts of the chicken industry from production to distribution level. Each participant was contacted by the first author and briefed about the project. If they agreed to participate, interviews were conducted.

**Table 1:** Type and number of participants interviewed.

**Type of participant**	**Broiler production**	**Layer production**
Federal and provincial poultry research officials	1	1
Focus group discussion with independent broiler farmers (*n* = 9)	1	0
Managing director of fully integrated company	1	0
Production managers of partially integrated companies	2	2
Independent chicken growers (environmentally controlled)	4	3
Independent chicken growers (semi-environmentally controlled)	0	2
Backyard farmers	0	2
Chicken and egg traders	3	2

### Data Collection

Qualitative data were collected using an interview guide that included questions on production system types, sourcing inputs, output distributions and chicken disease management (Supplementary Material 1). At the same time, quantitative data on the proportions of market share and value chain flows were collected. The identities of all participants were anonymized to comply with ethical and business confidentiality requirements.

#### Scoping Interviews

Initially people with extensive experience and knowledge of the chicken (broiler and layer) industry and food systems were identified and approached for scoping interviews. The aim of these interviews was to gain a high-level overview of the chicken production, its structure, types, trading systems, and disease control systems. Additionally, these interviews were used to identify the major key informants (KI) and stakeholders involved in the chicken value chain, who could then be subsequently contacted for more detailed interviews.

During the scoping interviews, participants were asked to: (1) describe the different production systems in terms of purpose, species, management, husbandry (including housing), and number of birds; (2) estimate the proportions of different poultry production systems in Pakistan; (3) provide an overview of the value chain nodes in the chicken production systems including identification of stakeholders and key markets or infrastructures.

#### Key Informant Interviews and Focus Group Discussion

Following the scoping study, the KIs or stakeholders identified by the participants of the scoping interviews, were interviewed using semi-structured interviews (Table 1). A FGD (*n* = 1) with nine independent broiler farmers was initially conducted, but this approach was replaced by face-to-face interviews with key informants (*n* = 23), as the FGD was perceived to be inefficient and unproductive due to cultural dynamics.

During the FGD and KIIs respondents were asked to describe the following: (1) flock size and type of birds; (2) sources of inputs (feed, vaccination, veterinary services); (3) types and distribution of outputs (live chicken, meat, eggs, manure); (4) types of people involved (buyers, retailers, brokers, traders); (5) flows of inputs and outputs and their association with one another; (6) amount of different outputs obtained; and (7) institutions and people involved in disease reporting, control and management.

Interviewees were asked open-ended questions (e.g., “what are different ways of distributing outputs from chicken farming?”). Various prompts were used to explore and clarify details on activities, people involved and product flows. The participants were asked to describe and discuss people, inputs, outputs, flows, and quantities in the system. During this process, the interviewer drafted flow charts that participants could clarify and amend. This iterative process was followed to create a preliminary map of the system by the interviewer; it was subsequently checked and approved by the interviewee. All interviews were conducted in Urdu language by the principal author and were audio recorded. Additionally, summary notes were taken of all discussions held. The complete question guide is available in Supplementary Material 2.

### Data Analysis

Through careful listening of the audio recordings the data were translated and transcribed. The notes and flow charts taken during the interviews and the FGD were then added to the transcribed recordings in a Microsoft Word document which allowed initial familiarization with data and preliminary structuring of information.

Analysis was done in two parts: first, a mapping analysis was performed to assist in the creation of flowchart diagrams, building on the drafts from the KIIs. This step allowed the creation of mapping profiles for different sections of the broiler and layer systems. These showed the type of people involved, flow of inputs, outputs and other chain characteristics that are key components of broiler and layer production systems. Where possible, proportions, or sizes were indicated as integers and with arrows of variable widths, according to the magnitude of the flows.

Secondly, thematic analysis was performed to identify meaningful themes that would provide understanding of the processes, governance, and interactions within the chains. Data were imported from Word into NVivo software (NVivoPro, version 11) and coded and arranged on the basis of similarity of information in the codes. Subsequently, the various themes were identified based upon codes that described an activity or characteristic of a value chain node. This thematic analysis was used to refine the mapping profile generated in the first step. Every time an interaction, stakeholder or activity was mentioned as associated to a particular chain, this was added to the mapping flow-chart diagram. Based on the broad topics asked during interviews, key themes of governance in the form of dominance, management, health provision, and identification of structural deficiencies in the poultry sector were identified and coded. All categories and themes were proof-read by co-authors as a quality check to avoid any gaps in theme identification and categorization.

## Results

### Structural Components and Types of Chicken Farming

Respondents described that over 75–80% of chicken meat and egg production in Pakistan was commercial in environmentally controlled intensive systems, while backyard birds accounted for 20–25%. Keeping chickens in the backyard was described as subsistence to meet household meat and egg consumption using free range systems. Three production systems for broilers and two for layers were identified (Table 2). These systems include: Fully integrated production (only for broilers), partially integrated production, and independent farming (**Figures 2**, **6**). Fully integrated producers were those where one single company managed the value chain, from grandparent production to finished chicken products sold to retailers or consumers. Partially integrated producers included companies that managed parent stock/breeder, finishing of broilers or layers, with varying control of the distribution of products or finished birds (but no grandparent stock). Independent farmers were described as broiler and layer growers who practiced rearing of day-old chicks (DOCs) to the level of finished birds in the case of broilers, and to egg production in the case of layers.

**Table 2:** Main characteristics of broiler and layer production systems.

	**Type of** **integration**	**Grandparent** **stock (GP)**	**Parent** **stock (PS)**	**Grower and** **production farms**	**Housing**	**Feed** **source**	**Veterinary** **services**	**Contracts**	**Value** **chain**	**Processing** **and packing**	**Market** **type**	**Export**
Integrated broiler production	Fully integrated broiler production (FIBP)	Owned	Owned	Owned broiler grower farms	Environmentally controlled	Owned	Privately hired	No actors at this point	Owned	Owned	Processed market	Processed meat
	Partially integrated broiler production (PIBP)	No actors at this point	Owned	Owned and contractual broiler grower farms	Environmentally controlled	Owned	Privately hired	With IBP and middlemen	Owned and middlemen moderated	Owned	Processed and wet market	Live birds and processed meat
Independent broiler production (IBP)		No actors at this point	No actors at this point	Owned broiler grower farms	Environmentally controlled and open-sided house	Owned and commercial feed	Privately hired and public services	No actors at this point	Middlemen moderated	No actors at this point	Wet market	Live birds
Integrated layer production	Partially integrated layer production (PILP)	No actors at this point	Owned	Owned layer grower farms	Environmentally controlled	Owned	Privately hired	Yearly contracts with target customers	Owned and middlemen moderated	Owned	Processed and wet market	Eggs and spent hens
Independent layer production (ILP)		No actors at this point	No actors at this point	Owned layer grower farms	Environmentally controlled, semi-environmentally controlled and open-sided house	Owned and commercial feed	Privately hired and public services	Yearly contracts with target customers	Middlemen moderated	No actors at this point	Wet market, retail outlets	Spent hens
Backyard farming (BF)		No actors at this point	No actors at this point	Rear birds in backyards	Free range	Mainly scavenging	Public services	No actors at this point	Middlemen moderated and direct sale	No actors at this point	Wet market	No actors at this point

### Broiler Systems

#### Characteristics of Broiler Farming Systems

**Figure 1:**
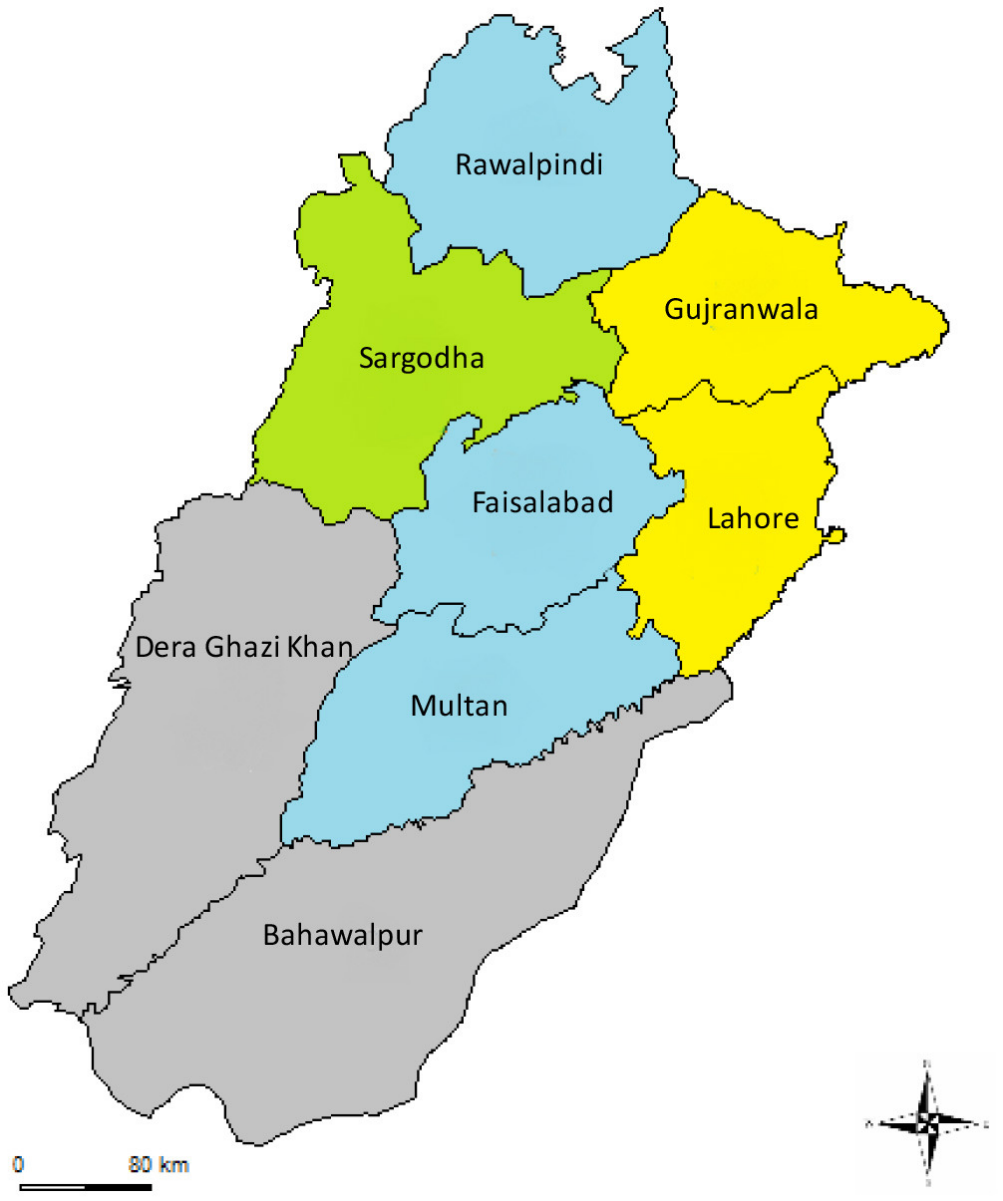
Major (colored) and minor (gray) commercial chicken producing areas of Punjab, Pakistan. Yellow: major broiler producing areas, green: major layer producing areas, blue: both broiler and layer producing areas. Map has been downloaded from https://gadm.org/maps/PAK/punjab_2.html and modified according to our data.

Commercial broiler farming management is divided into environmentally controlled and open-sided house systems. Major broiler producing areas in Pakistan were reported to be in Punjab, in particular in the districts of central and north Punjab (Figure 1).

**Figure 2:**
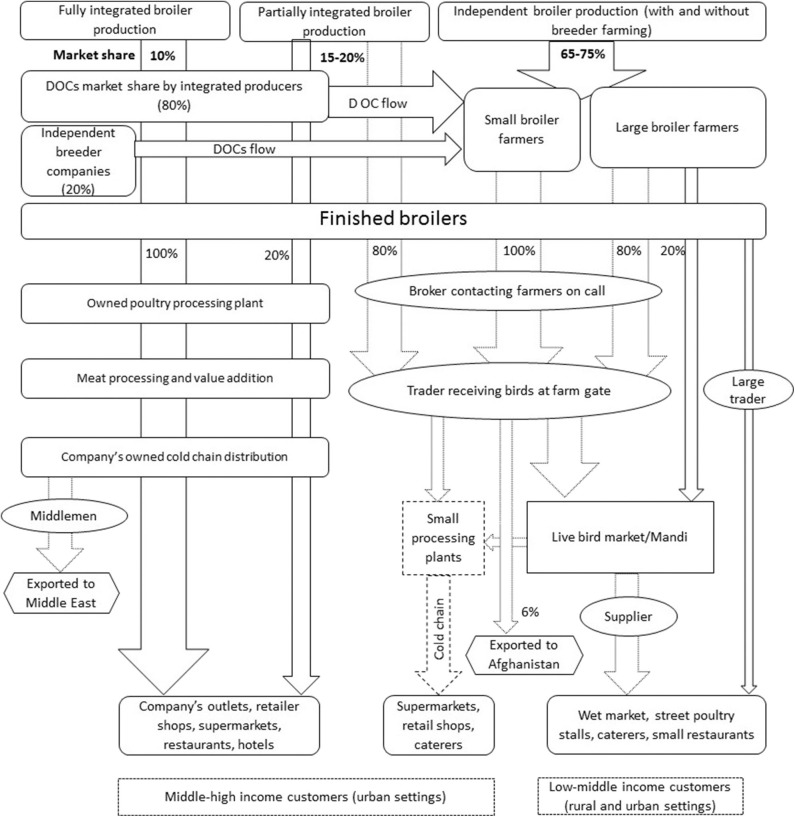
Broiler production systems in Pakistan, their market shares and associated value chains. DOC indicates day old chicks, Mandi in local language Urdu is a wholesale live bird market where birds are sold by open auction. Dotted lines show the parts of chains operated by the middlemen. Numbers in and around arrows and width of arrows indicate the market shares.

Major production system with their value chains are shown in Figure 2. Participants estimated that 97–98% of broiler commercial farming in Pakistan is within environmentally controlled houses characterized by automatically controlled temperature, humidity, feeding and water supply. Housing capacity is around 30,000–40,000 birds per house, with an average live weight of 1.5–2.0 kg per bird at the end of production cycle and a feed conversion ratio (FCR) of 1.2–1.6. Open-sided house broiler systems were reported to be used in <2–3% of all broiler production. In these systems birds are kept in open-sided houses (2,000–5,000 birds per house) with no control over the temperature of the house and manual provision of feed and water. Open-sided house farming was perceived to be decreasing rapidly due to the “increased risk of disease outbreaks,” “poor management of birds under extreme weather conditions,” “increased mortality rate,” “poor feed conversion ratio,” and the “reduced number of finished birds” produced in this system.

#### Types of Broiler Birds

Key informants reported that Arbor acres, Hubbard, Cobb, and Ross are the broiler breeds most often used in Pakistan. It was perceived that Hubbard breed was more popular in the past, but has lately been replaced by Cobb accounting for 50% of total broiler production. Hubbard, Ross and Arbor acres were estimated to represent 25, 15, and 10%, respectively. This shift from Hubbard to Cobb was believed to be due to a change in farmers' and consumers' preferences. The major reasons included “better FCR,” “comparable quality of meat,” and “increased amount of breast meat per bird.” Breast meat was reported to be directly related to profit gained, and hence preferred.

#### Mapping of the Fully Integrated Broiler Production Systems (FIBP)

**Figure 3:**
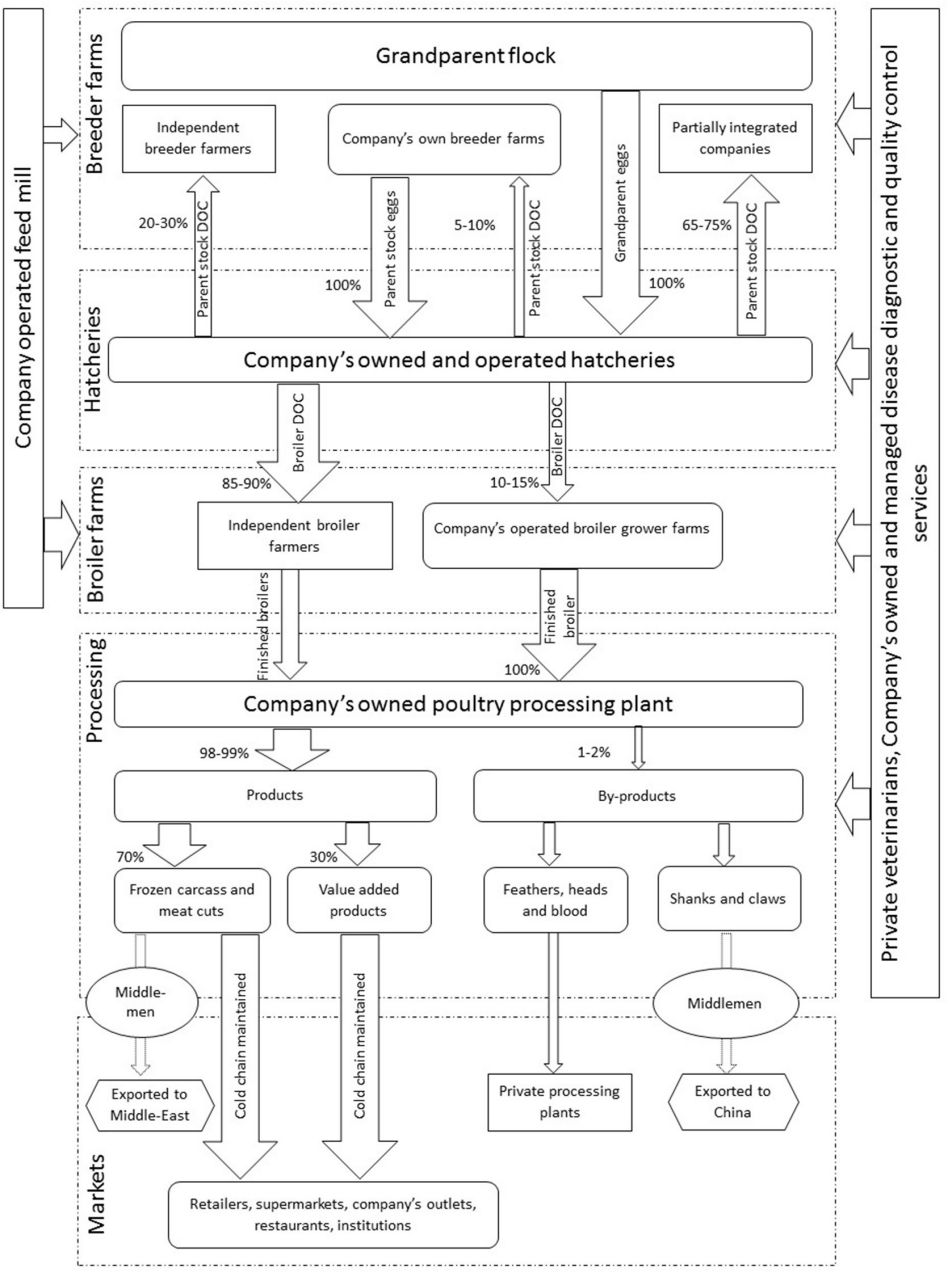
Map of the fully integrated broiler production system with distribution chains and target markets. DOC indicates day old chicks, numbers in and around arrows and width of arrows indicate the market share. Dotted lines show the parts of chains operated by the middlemen.

These systems were characterized by single ownership of the entire value chain (Figure 3). There were two fully integrated companies in Pakistan each with 1.5–2 million broiler DOC capacity at any one time. Their contribution toward total broiler production in Pakistan was estimated at 10%. These companies were reported to import and breed broiler grandparents to produce broiler parent stock. These parent stock DOCs were supplied to commercial broiler breeders (20–30%), partially-integrated broiler companies (60–75%), and to their own parent breeder farms (5–10%). The latter produced commercial broiler DOCs supplied to their own farms (10–15%) and to independent broiler farmers (85–90%). Interviewees explained that these companies owned feed mills used to provide feed not only to their own farms (breeder and broiler grower farms) but also to sell commercially. This was reported to be one of the most profitable commodities in integrated chicken production.

Central management of these companies' broiler growers was reported to ensure good farm practices and quality of the finished product, through maintaining environmentally controlled systems and strict biosecurity protocols. Company-operated vehicles were said to transport finished birds from their grower farms to processing plants that are mainly located on the outskirts of Lahore city, with the capacity to process a maximum of 50,000–60,000 birds per day. It was also reported that these companies are Halal (ISO 9001) and international food safety management system (ISO 22000) certified and operate using a hazard analysis critical control point system. Chickens were processed into frozen carcasses, meat cuts and ready to cook products (98–99%), with 1–2% by-products that included shanks with claws, feathers, intestines and blood. Shanks with claws were exported to China while other by-products were sold to commercial rendering plants. Finished products were supplied to the company's own outlets and to independent retail grocery shops, supermarkets, restaurant chains, and various institutions like hotels and clubs throughout Pakistan via company-operated refrigerated vehicles.

Interviewees explained that the processed products were mostly used domestically in the country, although frozen carcasses and ready-to-cook products were also exported to Middle Eastern countries like Qatar, Abu Dhabi and Bahrain. Export was described to be the only part of the FIBP value chain moderated by middlemen. In case of high demand of processed chicken from target customers, these companies were reported to purchase broiler birds from independent broiler farmers. Such deals were described to be devoid of any middlemen involvement and required strict bird health criteria to complete the purchase, which include birds being negative for Salmonella, Mycoplasma infection, and free from antibiotic residues.

#### Partially-Integrated Broiler Production Systems (PIBP)

**Figure 4:**
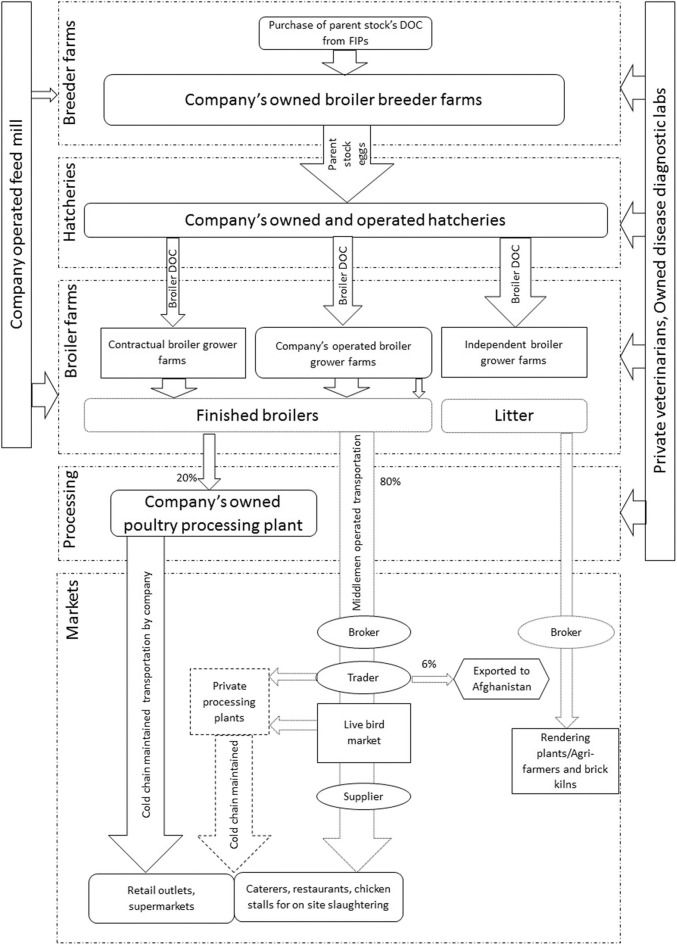
Map of partially-integrated broiler production system with distribution chains and target markets. DOC indicates day old chicks, numbers in and around arrows and width of arrows indicates the market shares. Dotted lines show the parts of chains operated by the middlemen. FIPs refers to fully integrated production system.

Operations of PIBP are shown in Figure 4. Respondents revealed that the market share of PIBP was 16–20% of the total broiler production in Pakistan. PIBP starts operation at the level of parent stock (breeder) farming. A minority of these companies were found to practice broiler parent stock farming but do not have their own distribution chains and processing plants, while the majority purchase the broiler parent stock DOCs from the FIBP. All of the PIBP were described to have their own broiler parent stock farms, own broiler grower farms, and own feed mills. The DOCs produced were sold to either company-owned broiler grower farms, independent farms or contractual farms. Contractual farms are those with long-term supply contracts; they are bound to buy broiler DOCs and feed from PIBP in return for animal health services and purchase of the finished birds. All housing was environmentally controlled with an average of 30,000–40,000 broiler birds per house and 4–5 broiler houses per farm.

The PIBP broiler value chain was found to include either informal middleman-governed distribution chains, or formal company-operated distribution chains. The respondents explained that the majority of finished broilers (80%) were sold as live birds to designated brokers who have contracts with the companies. These brokers have additional contracts with traders to collect finished birds from the farms and supply them to live bird markets (“Mandi” in local language Urdu; a wholesale live bird market where birds are sold by open auction) or independent poultry processing plants. The remaining 20% of the finished broilers produced were said to undergo processing to finished broiler products via company-operated processing plants followed by transport via company-designated refrigerated vehicles to local restaurants, grocery outlets, supermarkets, and the companies' own outlets in the country.

The PIBP as explained by the interviewees were in transition from independent broiler farms toward a fully integrated system. The reasons reported for this transition were the “unstable live broiler market,” “price fluctuations due to seasons and festivals,” and to “bypass middlemen” dependency in the distribution chains.

#### Independent Broiler Production (IBP)

**Figure 5:**
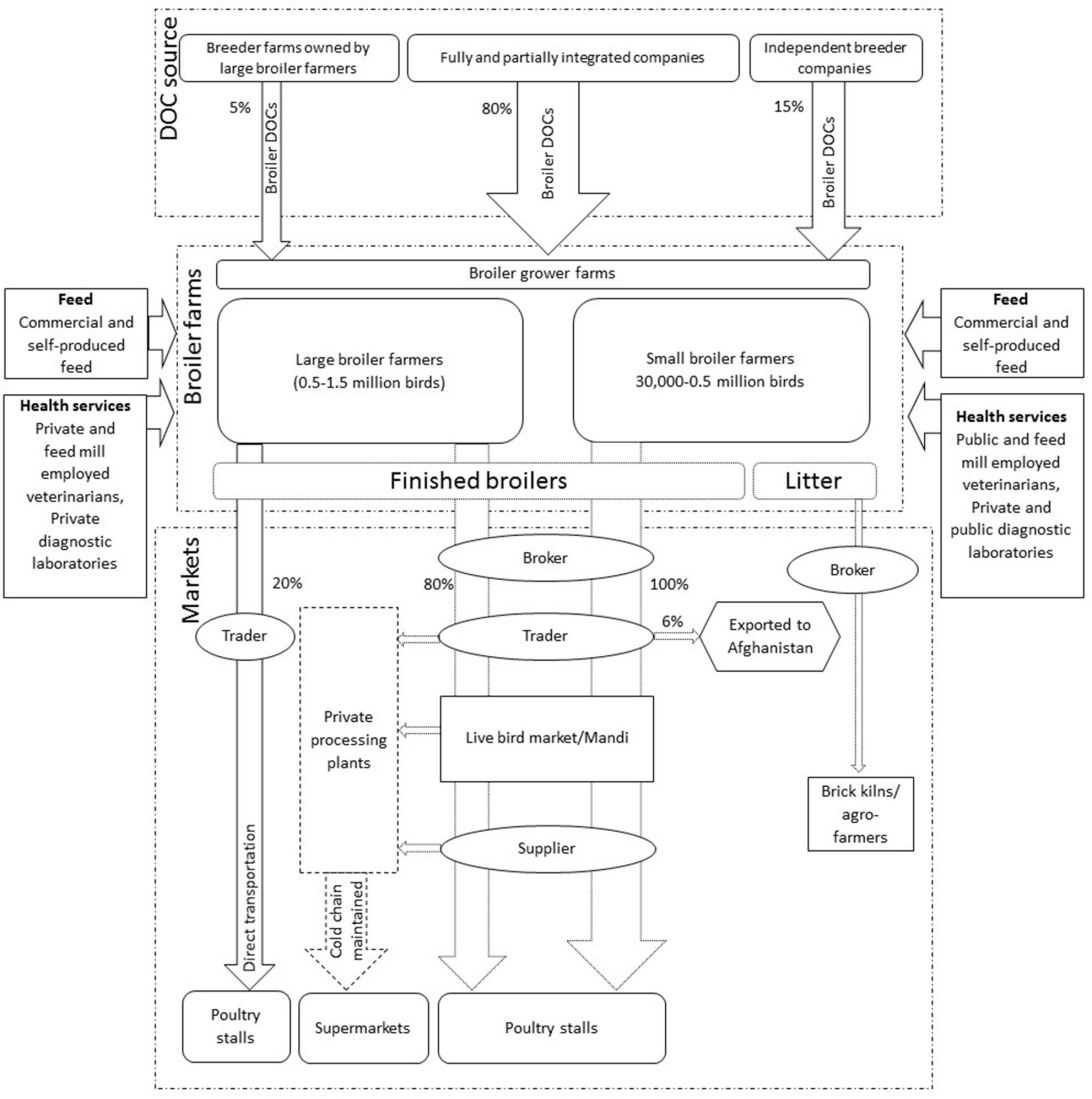
Map of independent broiler farming, its types, distribution chains, and target markets. DOC indicates day old chicks, Mandi in local language Urdu is a wholesale live bird market where birds are sold by open auction. Dotted lines show the parts of chains operated by the middlemen. Numbers in and around arrows and width of arrows indicate the market shares.

It was estimated that independent broiler farming (Figure 5) is a major contributor (65–75%) to the number of finished broilers produced in the country. The farmers in IBP were only involved in the raising of broiler DOCs to the level of finished broiler. Most of the major inputs such as feed and DOCs were said to be purchased directly either from fully or partially integrated companies, or from independent feed mills and hatcheries. Finished broilers were sold to the brokers at the farm gate as live birds. Output distribution chains in IBF were mainly regulated and controlled by middlemen including brokers, traders, suppliers, and retailers.

Farm level decisions in IBP such as selecting breed of broilers, sourcing feed, and vaccination were reported to be made by either the farmer, farm manager or farm supervisor. These decisions were reported to be autonomously made without being influenced by any FIBP or PIBP, despite them being major providers of DOCs and feed to independent farmers.

##### Types of independent broiler farming

Independent broiler production was further categorized into large and small scale depending on total housing capacity. Small scale broiler farmers were described to have capacity to house between 30,000 and 0.5 million broiler DOCs, while large scale broiler farmers could house between 0.5 and 1.5 million birds; very few farmers were reported to have capacity for >1.5 million broiler birds. To reduce the cost of production and increase efficiency, it was reported that 85–90% of IBP is within environmentally controlled systems. The major input costs as perceived by the farmers were for feed (60–65%) and DOCs (25–35%), followed by vaccination and medicine (12–15%). Feed and DOCs were provided directly to the farm level by the independent feed millers and breeders, FIBP, PIBP via company-operated vehicles without the involvement of any middlemen. Large scale broiler farmers tended to produce their own feed with extra feed purchased commercially if needed. In contrast, small scale broiler farmers were reported to depend on commercially available feed, mostly supplied on a credit basis. The majority of DOCs (80%) supplied to these independent farmers is provided by 10–12 companies (FIBP or PIBP), and their distribution offices were reported to be located around the major poultry producing areas, such as central and north Punjab. These companies have 80% of the country's parent stock flocks, while the remaining 20% parent stock is distributed among small and large independent broiler breeder companies.

##### Distribution and value chains in IBP

Numerous chains with middlemen, live bird market and wholesale markets were found for the sale of finished broiler within IBP (Figures 2, 5). These distribution chains were dominated by three types of middlemen, namely brokers, traders, and suppliers. A broker was defined by the respondents as an agent that deals with farmers and traders in order to purchase finished broilers. They were found to be actively communicating with the local farmers and traders to negotiate deals regarding the farm gate price of finished broilers. A trader was defined by informants as a person that purchases birds on credit from a broker, gets commission, and transports birds from farms to poultry wholesale markets. Informants explained that the sales of birds at the level of farmer and broker were cash based, whereas sales between broker and trader were mostly credit based. Suppliers (“gari wala” in Urdu means a person with a vehicle to transport birds) were responsible for transporting birds from live bird markets to the poultry stalls but in less capacity compared with traders; these purchases were in cash. Poultry stalls or shops are the commercial premises where live birds are kept and halal-slaughtered per demand. In northern Punjab, including districts of Chakwal and Rawalpindi, some large-scale farmers were reported to practice direct transportation of finished broiler to the Mandi to bypass brokers and traders, but such farmers also depend on open auctions conducted by traders in live bird markets to sell their products.

#### Broiler Chicken Marketing Profiles

Independent broiler farming (IBF) was described as a major contributor to Pakistan's broiler production. Live bird markets serving this production system dominate; they link brokers, traders and suppliers in the sale of finished birds (Figure 5). Farmers expressed distrust toward these middlemen for creating price fluctuations of finished birds and eggs at farm gate and market level. The live bird markets were described as wholesale markets where live birds from various sources were aggregated via middlemen and further distributed to retail outlets. These markets were described to be mostly operational in big cities, like Tollinton and Sheranwali market in Lahore, or in high chicken producing districts of the country. These markets also supply chicken to independent poultry processing units situated around big cities. In small cities, brokers and traders purchase birds directly from the farm and supply them to retailers (i.e., chicken stalls/butcheries).

To ensure halal slaughtering and respond to consumer preference for freshly slaughtered meat at cheaper rates, wet chicken markets in the form of retail outlets (chicken stalls) were described to be more common than other sources. These stalls were distributed all over the country in and around residential areas, serving freshly slaughtered halal meat daily based on demand. They were reported as the major provider of chicken meat to low- and middle income customers. Conversely, the FIBP and PIBP supplying a wide range of value-added products tend to target high-income consumers by selling these processed products at a higher price in more formal settings and ensuring that food safety standards are met.

### Commercial Layer Farming

**Figure 6:**
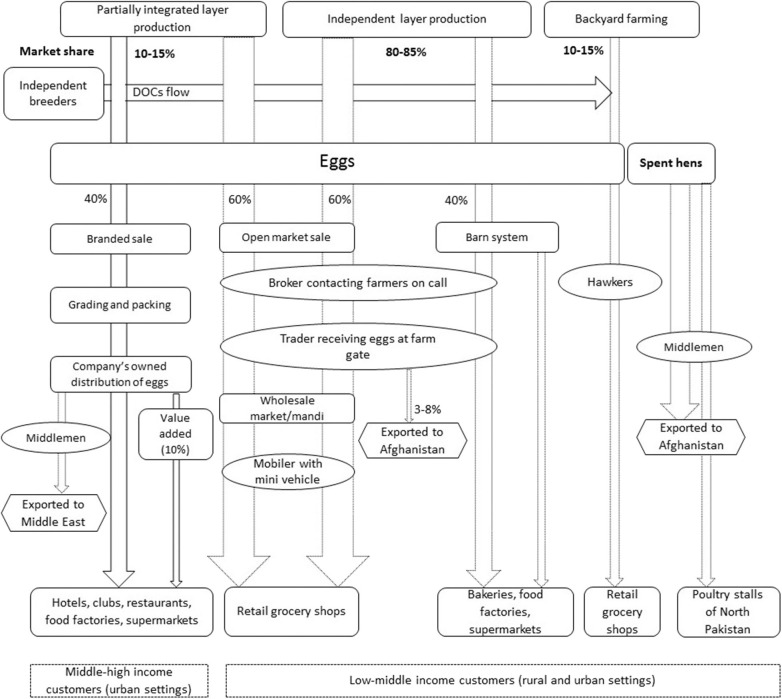
Layer production systems in Pakistan, their market share and associated value chains. DOC indicates day old chicks, Mandi refers to the wholesale market for eggs. Dotted arrows show the chains operated by the middlemen, numbers in and around arrows, and width of the arrows indicate the market shares.

Figure 6 shows the major layer production systems in Pakistan. Respondents described layer farming as a growing sector in Pakistan, transforming rapidly from conventional to modern farming practices in order to cater to the increased demand for eggs and egg-based food products. It was reported that 60–70% of the layer farming was environmentally controlled (as described in section Characteristics of Broiler Farming Systems) while the remaining 30–40% were open-sided housing systems. Layer farming was perceived to be in transition from the conventional open-sided house with floor rearing system to modern environmentally controlled cage systems. Informants respondents said that almost 80 million layer birds were reared in cages, almost 20 million in locally produced cages and 117 million on floors in open-sided houses.

Respondents stated that layer production is less profitable than the broiler production due to; a longer production cycle creating seasonal placements (February–March) of layer DOCs in rearing houses, high feed costs, and a lower probability (33%) of getting female chicks out of hatched eggs. A total of 40,000–50,000 layer breeders were present in Pakistan; they were distributed among 7–8 layer breeder companies and big layer production farms. Layer farming was mostly practiced as independent farming or as partially integrated layer production. Processing of table eggs was not reported to be commonly practiced and only one independent company throughout the analysis was reported to process eggs into egg powder and packed liquid eggs.

#### Types of Layer Birds

The major layer breeds in Pakistan were Hy-Line (W-98, W-36, CV-22), Babcock, Lohman (LsL light, LsL classic), Novogen (white light, brown light), H&N international (nickchick, crystal nick, coral brown) and Hendrix genetics (Shaver, Bovan and Hisex). LsL light was believed to be the most popular breed in Pakistan due to “high egg production efficiency” and its “fitness for the cage system” because of its light weight. Average peak production of commercial layers in Pakistan was described to be reached at the age of 26–29th weeks with an average number of 320 eggs produced per bird per unit production cycle. Major layer producing areas reported in Pakistan included districts of central Punjab (Kamalia, Arifwala, Okara, Sammundri, Sargodha, and Faisalabad), north Punjab (Chakwal and Rawalpindi) and south of Sindh province (Karachi and Hyderabad) (Figure 1).

#### Partially Integrated Layer Production System (PILP)

The interviews revealed that no fully integrated layer production system exists in Pakistan (Figure 6)*;* there were no reports of production and breeding of layers' grandparents in the country. The layer parent stock is imported from United States and Europe, and kept either by partially integrated companies or by independent breeders. Companies were designated as partially integrated due to their absence of keeping layer grandparent and partial ownership of distribution chains (40%). All major inputs like feed and DOCs were reported to be provided by company-owned feed mills and hatcheries.

The market share of egg production of PILP in Pakistan was estimated to be around 10–15% (Figure 6). Only two layer companies were reported to be partially-integrated with environmentally controlled cage systems (Table 2) for layer rearing and production; owning their feed mills, and diagnostic laboratories. Both were involved in grading and packing of eggs and company-operated distribution of the eggs to the target customers.

The partially-integrated layer companies had their own egg distribution chains, called “branded sale” (40%), while the rest of supply was moderated by middlemen (60%). Branded sale was reported to include grading and packing eggs before distributing it to hotels, clubs, restaurants, supermarkets, retail shops, and company owned outlets via company-operated vehicles. Moreover, such companies were also reported to add value to eggs by Omega 3 enrichment and deeply-pigmented yolk in line with consumer preferences. These value-added eggs were only 10% of all eggs produced by partially integrated companies.

#### Independent Layer Production (ILP)

The market share of ILP was about 80–85% of the total egg production in Pakistan (Figure 6). Only a few farmers kept layer breeders in ILP and the majority purchased DOCs either from independent breeders or PILP. Farmers may or may not have their own feed mills in case of ILP. It was reported that 70% of the ILP was environmentally controlled farming (as described in the section Characteristics of Broiler Farming Systems) while 20% was semi-environmentally controlled where temperature and humidity were controlled only in summer with minimum ventilation in winter by manually regulating the house vents. The share of open-sided house farming was reported to have reduced from 40 to 8–10% in the last decade. This reduction was believed to be due to increased competition for better quality eggs, and increased consumer demand of eggs. The interviewees predicted that the remaining open-sided house layer farms will be completely replaced by semi or completely environmentally controlled farming in the coming years.

In open-sided house based farming layer birds were kept on the floor during the production phase with manual egg collection performed 4–5 times a day, in contrast to cage production where all eggs were automatically collected once a day, thereby reducing labor costs. In ILP, 60% of the eggs were reported to be traded through open markets dominated by brokers, traders and “mobilers” (Figure 6). Mobilers were described as traders who distribute eggs to the retail outlets via motorcycle or mini vans, similar to suppliers in broiler distribution. Moreover, 40% of eggs were traded through the so-called “barn system” in which a yearly contract of egg supply was either signed with big traders, bakeries or food production companies. Spent hens in PILP and ILP were reported to be exported as live birds to Afghanistan or sold to northern hilly areas of Pakistan via traders.

### Backyard/Chicken Production

Backyard production in Pakistan was defined as keeping 2–3 birds in the backyard of a house for recreation or domestic use. Backyard production was predominantly found in remote areas of Pakistan where it is difficult to maintain a continuous supply of inputs. The backyard birds were reported to be mostly kept in rural areas to meet household needs of eggs and meat with surplus eggs and spent birds sold to hawkers. Hawkers were said to sell these birds to local markets/retailers and wholesale markets of big cities like Lahore. The breeds that were kept for backyard farming included Desi, Fayoumi, Rhode Island Red, Naked neck, and their crosses. The housing system was free range and feed sources included scavenging and kitchen by-products. Major areas of backyard farming included rural areas of Chakwal, Mianwali, Bhakkar, and Dera Ghazi Khan Districts of Punjab. As the backyard farming was scattered through secluded rural areas of Pakistan, backyard farmers were reported to have only access to health facilities provided by rural government veterinary or para-veterinary staff.

### Dynamics of Chicken Markets

Interprovincial unregulated transport of chicken was evident in the study. The production surplus in Punjab province, along with high production prices in other provinces were described as major factors that caused movements of birds from Punjab to other provinces. Some participants explained that traders from other provinces acquired birds from Punjab if there was enough profit margin left after deducting transportation cost and weight loss during transport. The price difference of broiler meat between Punjab and other provinces was estimated at 40 PKR/Kg (0.26 USD/Kg) live weight and farmers reported that traders from other provinces traveled a distance of 900–1000 Km toward central Punjab to purchase live birds at a cheaper rate.

Respondents described cross-border trade with Afghanistan for exporting finished broilers, spent hens and table eggs from Pakistan. Due to a lack of import standards in Afghanistan these exports were described to be free of any safety checks and quarantine procedures. This trade was reported to be moderated by the traders on either side of the border based on credit or cash depending on the type of agreement. However, the respondents showed concerns about “cash recovery” in this trade.

Commercial broiler and layer producers were located close to urban settlements to allow easy access to the markets. A rapid increase in using processed meat and ready-to-cook chicken products by consumers was reported. This was mainly due to increased number of large- and small-scale slaughterhouses around the major poultry producing areas of Punjab province. Improved meat processing technologies, good hygienic practices, and strong marketing tactics, with electronic and print media used to increase consumer awareness about the safety and hygienic food, were the major reasons reported for increased consumption of chicken products. Such marketing campaigns were moderated by meat and egg processors mostly targeted at medium-high income customers as low-income groups cannot afford to pay 50–100 PKR (0.5–1 USD) extra for the same weight of chicken that they could easily get from informal chicken stalls. The situation was described to be similar for egg marketing. Respondents also hypothesized that with increasing demand of ready-to-eat products and ease in household handling of processed meat these markets could overtake wet markets in the future.

### Health Services Providers and Structural Deficiencies Identified in Chicken Sub-sector

The availability of poultry health services varied among different chicken production systems. Two main types of stakeholders (Table 3) were reported to provide health services to farmers, namely those in the government (70%) and private (30%) sectors. The government sector was found to be actively involved in passive surveillance of diseases like AI, providing some vaccines and capacity building against chicken diseases while the private sector was involved in providing vaccines and diagnostic services. “Trust in quality,” “price of available services,” and “easy access to the health services” were stated as a major factors in selecting available diagnostic services (government/private laboratories) and control measures (local/imported vaccines). Backyard farmers and small-scale independent farmers relied on government veterinarians, feed, and medicine company veterinarians, experienced para-veterinary staff as well as government and small private laboratories for obtaining bird health services. Vaccines used in these systems were mostly locally produced. Commercial chicken farmers in FIBP, PIBP, PILP and large-scale independent farmers tended to hire their own private veterinarians along with visiting experienced private consultants and used well-established private and government laboratories in big cities for disease diagnosis. This is because of their large farming setups and desire to ensure good quality finished products through regular monitoring of bird health. Some FIBP and PIBP reported having their own diagnostic laboratories for disease diagnosis, including for AI, which also provided commercial services to independent farmers. The integrated producers also reported a lack of interest in the government provided health and diagnostic facilities and vaccines, having concerns regarding quality of available services. The vaccines for AI and other poultry diseases mainly used by these farmers were either privately produced locally or imported from Europe and China. In general, layer and broiler farmers thought that vaccines were necessary to prevent and control infectious diseases like H9N2 AI.

**Table 3:** Major themes identified regarding management and health services by production system.

**Themes**	**Sub-themes**	**Full integration**	**Partial integration**	**Independent production**
Sector level management	Dominance	Day old chicks supply, Feed supply, Processed market	Day old chicks supply, Feed supply, Processed market	Wet market, Over all chicken meat and egg supply, Middlemen
	Price fluctuations	Slightly concerned, Economic instability	Moderately concerned, Economic instability	Highly concerned, Economic instability, Middlemen monopolization
	Role of poultry association	Farmer meetings, Disease control strategies	Farmer meetings, Disease control strategies	Farmer meetings, Disease control strategies
	Role of government	Poultry Production Act, 2016	Poultry Production Act, 2016	Price management Poultry Production Act, 2016
	Inter-farm distance	Highly concerned	Highly concerned	Moderately-highly concerned
Farm level management	Biosecurity	Strict biosecurity	Strict biosecurity	Variable biosecurity
	Labor staff	Technical and experienced	Technical and experienced	Non-technical and experienced, Technical and experienced
	Dead bird disposal	Burying, Burning	Burying, Burning	Burying, Burning, Throwing on landfills and water bodies
Animal health	Private services	Veterinarians, Vaccines, Diagnostics, Medicine supply	Veterinarians, Vaccines, Diagnostics, Medicine supply	Veterinarians, Consultant veterinarians, Vaccines, Diagnostics, Medicine supply
	Government services	No role	No role	Veterinarians, Vaccines, Diagnostics, Passive surveillance

Medicines and vaccines were reported to be purchased directly from regional distributors or veterinary pharmacies, and veterinarians were found to be involved in their marketing and sale. These veterinarians also provided free health consultancies to independent chicken farmers. Only one company (PIBP) was found to operate their own pharmaceutical units producing medicine including antibiotics for their own farming business and for commercial sale. In case of any notifiable disease outbreak, especially high pathogenic AI at the district level, it was the responsibility of the local government veterinary officer to inform the assistant disease investigation officer, who further informed the divisional disease investigation and control officer followed by provincial and federal reference laboratories. The Pakistan Poultry Association was found to be the major organization actively working as a link between government and poultry industry to address the issues of the poultry farming community at federal level. At provincial and federal level, strategies were developed and updated in consensus with Pakistan Poultry Association under the umbrella of Poultry Production Act, 2016, to devise and disseminate information on disease control and interventions. This included restriction of animal movement, adoption of strict control measures and increased surveillance in the affected areas. However, for low pathogenic AI H9N2 virus no special reporting system was stated during the interviews. In FIBP, PIBP and independent large-scale farming, internal disease reporting systems, including H9N2 AI infection, were reported that did not involve government veterinarians. Most of the reported H9N2 AI outbreaks in Pakistan happened between the months of March–April and October–November. The farmers were aware of such seasonal outbreaks and “mentally prepared” for losses during H9N2 outbreak months.

Respondents in all integrated and large-scale independent chicken production systems described burying or burning diseased and dead birds. Most of the small-scale independent farmers reported disposing of birds on landfills and in canals located close to their farms. The large-scale farmers showed concerns that such improper disposal and lack of regulations from government on disposal practices were major reasons for repeated outbreaks of H9N2 AI. Structural deficiencies as reported by the respondents during interviews are presented in Table 3.

## Discussion

The aims of this study were to characterize and map chicken production systems, associated value chains and to explore the options for chicken disease control in Pakistan. To the authors' knowledge, this is the first study to provide a detailed characterization of the chicken production systems and their value chains in Pakistan.

The mapping of the chicken production industry identified important differences in production types, chain structures, and marketing of finished products. The chain structure varied in terms of length and intricacy across profiles. Short chains were present in FIBP and some PIBP setups, while long chains were mainly found in independent production systems, where middlemen like brokers, traders, suppliers and mobilers were not only involved in distribution of products, but also in price control. While price control may be beneficial for single actors or a group of actors, it increases transaction costs and contributes to inefficiency within poultry value chains; this is in line with similar findings in other low and middle income countries (18–20). It was found that independent farming for broilers and layers was completely dependent on brokers and traders for selling finished chicken meat and eggs. Further down the distribution chain these middlemen relied on being able to sell live birds and eggs to wholesaler markets; a pattern also reported for Bangladesh (21). Control by middlemen was predominant in wet markets where price setting was used as a mechanism to influence supply and demand of chicken and its products. These findings are in accordance with studies in India (22) which associated high prices and inaccuracies in supply and demand with the presence of middlemen in poultry value chains. Despite the increase in transaction costs and middleman monopolization in the supply and demand of chicken and its products, the majority of farmers preferred to sell to brokers and traders at the farm gate because this cash-based sale was most convenient for them. This was the dominant type of transaction for partially-integrated and independent farming. It was similar to other studies conducted in Africa where farmers engage themselves in selling finished products at farm gate level to access cash quickly and to avoid transportation costs (23, 24). In these situations, ethics and attitudes of middlemen have the potential to influence the price of the finished product disproportionately (21). Complex distribution chains, with numerous middlemen, are known to limit profits to farmers (22, 25). For this reason, some farmers in north Punjab bypass middlemen by transporting and selling finished birds directly to the markets. Integrated companies on the other hand, for both layers and broilers, were involved in managing the whole value chain from the level of breeding stock to the distribution of finished products to ensure good quality of product and to reduce transaction costs. However, they have higher production costs due to applying strict hygienic measures for processing, value addition, managing transportation and advertisement costs. This results in a higher price of finished products (21), but they have better access to export markets and high end consumers.

People's perceptions about meat obtained from chicken stalls as fresh, halal, easy to access and cheap are factors causing wet markets to dominate. These findings are consistent with those of Karthikeyan and Nedunchezhian (26) in India who reported cheap prices of freshly dressed meat and accessibility of corner chicken/retail shops as major factors for preferring wet markets. Despite the high retail cost of processed products, a shift was perceived in consumer preference away from freshly slaughtered birds toward processed meat due to increased awareness about safe, hygienic, and value-added meat and eggs in Pakistan; as also reported in neighboring countries (26). This consumer shift could encourage integrated farmers to scale up processing operations and expand their business creating new potential for processed markets. However, similar to Bangladesh, Pakistani processed markets are not as popular as wet markets, as they do not have on-site and on-demand slaughtering (21). Moreover, the increase in the trend of processed meat consumption and the development of small and large private poultry processing units for catering has aided in the growth of domestic demand, and export of processed chicken products. Therefore, it makes financial sense to increase chicken production and processing in Pakistan as it could serve as a source of foreign exchange (27).

The chicken market in Pakistan (broilers and spent hens) is predominantly regarded as a live bird market and large independent and partially-integrated farmers were found to be involved in the export of live birds to Afghanistan–as previously reported (13). Farmers reported a lack of import standards for exporting live birds to Afghanistan and hence stated these exports as free from health and safety checks. However, such findings are not in accordance with Afghanistan poultry industry and import requirements (28) which details the criteria for importing chickens into Afghanistan.

Integrated companies in Pakistan were found to be involved in the export of processed and packed chicken meat and egg products including frozen carcasses, ready to cook items and value added meat and eggs; a practice reported earlier in India (26). However, these exports are minimal when compared with the vibrant domestic fresh meat and egg market. In 2012, total national production and consumption were approximately balanced at roughly 590 kilotons (13).

DOCs and feed in Pakistan are mainly supplied by integrated production systems, which is in accordance with findings from Kenya and Pakistan where dominance of large companies in supplying DOCs has been described (13, 18, 23). However, several small and large independent breeder farmers and feed mills, working in parallel with integrated companies have created a competitive market for DOCs and chicken feed across the production systems. Independent farmers in Pakistan were reported to be autonomous in making farm-level decisions about sourcing of DOCs, feed, vaccines, and selling finished birds and eggs without being influenced by the big players of the chicken industry. However, such independence could create a lack of coordination, making it difficult for farmers to adjust production according to changes in demand. This lack of coordination could lead to uncertain markets for poultry meat and eggs and price fluctuations in the poultry sector in Pakistan (13). Lack of government regulations on price control and uncertain retail markets, as also found by Chaudhry et al. (13), were described to cause high variation in the prices of DOCs and finished chicken products (eggs and meat) throughout the year.

Poor farm management practices were reported in the study that could play a role in AI outbreaks on farms (29, 30). Strict enforcement of control measures such as biosecurity and vaccination at the national level would help to control and manage farm level endemic H9N2 AI outbreaks successfully (31), but no relevant regulation was described by respondents. Farmers also associated the high poultry population density of central and north Punjab (32), and less inter farm distances in poultry rich areas (33) with repeated disease outbreaks in the country. Moreover, live bird market trade patterns and a lack of control over birds' movement (34) was thought to create a niche for pathogens to thrive, resulting in repeated outbreaks of diseases like Newcastle disease virus and H9N2 AI virus infection. These findings highlight important gaps in poultry traceability that could be bridged in the future to devise a successful disease control program. Moreover, disruptions caused by poultry disease may create unfavorable environments for new investments and threaten the survival of small-scale farmers.

Private and public sectors were equally important in controlling poultry diseases including AI at country and farm level. These stakeholders could be targeted to inform policy making and develop robust approaches for disease control (35). The study revealed limited coordination between private and public sector stakeholders providing health services. Partnerships between private and public agencies are highly recommended by the World Organization for Animal Health (OIE) for effective animal disease control, by encouraging rational use of resources especially in lower and middle income countries with limited capital (36). Moreover, such partnerships could create opportunities to expand export markets for fresh, frozen and processed meat, eggs, and their products.

The current study has some limitations. The qualitative nature of the study means that a limited number of participants were interviewed. However, participants were carefully selected because of their extensive knowledge of (parts of) the poultry value chains and to ensure a broad representation of diverse stakeholders. Their views, although believed to be a good approximation of the chains structure and its working, may present some bias. We included participants from large corporate level to small backyard farmers, experienced consultants to farm veterinarians, and large and small poultry traders that helped to cover the major aspects of the poultry sector from wet to processed markets, local, and export markets and disease control options in various poultry settlements. Because of challenges related to social and cultural norms, only one FGD was possible and the remaining data were collected through KIIs conducted face to face. It provided a chance for participants to express their opinions freely. Proportions like market shares, country level shares obtained during data collection were merely based on approximations and personal perceptions of respondents and their average is represented in the results. By-product chains were not explored in depth, but a brief description of various by-products was included.

The mapping gives an in-depth understanding of the structure of the chicken value chains in Pakistan thereby providing a basis for epidemiological disease modeling. Such modeling could help to identify critical control points for interventions toward safe and sustainable food. Identification of actors across various levels of the value chain can be used in further research to investigate personal beliefs and behaviors in relation to control measures. Finally, information on the linkages and processes in these chains provide a starting point for detailed investigation of transaction costs.

## Conclusion

Detailed value chain maps and information on integrated, independent, and backyard production were used to characterize the chicken industry in Pakistan, and to highlight structural differences between broiler and layer production systems. The analyses revealed the dominance of specific stakeholders, actors and markets in supplying chicken and its products throughout the country. Processed markets were mainly captured by FIBP, PIBP, or PILC where the role of middlemen was negligible, while the wet market was dominated by independent farmers where middlemen influenced the pricing of goods and supplied live birds and eggs to chicken stalls and retail shops. Lack of efficient government policies on price control and farm biosecurity were reported to lead to price fluctuations and inefficient disposal of dead birds. The current study provides baseline information on chicken value chains in Pakistan and identifies factors causing disruptions in the operations of this sector, along with aspects that influence prices. It can be used as a basis for economic impact assessment of chicken diseases and the calculation of economic efficiency of vaccines in different production systems. Stakeholders identified could be targeted for devising policies and novel interventions for efficient control of diseases in the industry.

## Data Availability Statement

The datasets generated for this study are available on request from the corresponding author.

## Ethics Statement

Ethical approval was sought from and granted by the Social Sciences Research Ethical Review Board (SSRERB) of the Royal Veterinary College, UK (project reference: URN SR2017-1303). Prior to data collection, informed consent was obtained from all participants for participation in the study, audio recording and photography. The participants provided their written consent to participate in the study.

## Author Contributions

HA collected, analyzed data, and drafted and revised manuscript. BH and PA were directly involved in developing study design, data analysis, writing of manuscript, and critically reviewed all manuscript drafts. MI contributed toward initial project planning and grant winning (award numbers BBS/E/I/00007034, BBS/E/I/00007035, BBS/E/I/00007038, and BBS/E/I/00007039), technical support in data collection, and reviewed drafts of manuscript. TY contributed toward provision of logistical and technical support while collecting data in Pakistan and reviewed drafts of manuscript. All authors contributed to the article and approved the submitted version.

## Conflict of Interest

The authors declare that the research was conducted in the absence of any commercial or financial relationships that could be construed as a potential conflict of interest.
