# Supplementary material for: A Value Chain Approach to Characterize the Chicken Sub-sector in Pakistan
Source: Front Vet Sci. 2020 Jul 3;7:361. doi: 10.3389/fvets.2020.00361 (PMC7351015; doi:10.3389/fvets.2020.00361)
Supplement: Supplementary file 1 [file Data_Sheet_1.pdf]

Supplementary material 1

Questionnaire for scoping study interviews to the focal persons of chicken industry to assess chicken industry structure and trade patterns

Royal Veterinary College

Before starting:
Introduction
Description of project and purpose of the interview
Time for questions
Presentation of the consent form and acquisition of informant's signatures.

Questions
Section A: About role of your association or group

1. Could you tell me briefly about your role or the role of your group in the poultry industry?

Section B: Understanding poultry industry structure

For my project, I want to identify the different stakeholders and their connections and relevance for the poultry industry. This will help me to establish the type of farms that I could look at for assessing vaccination efficacy and the potential impact of avian influenza infection and vaccines in the industry.

2. Could you please describe the structure of the broiler industry and layer industry?

We have organized the interview in different steps of the value chain: breeder, hatcheries, poultry producers, feed companies, animal health services, traders/markets and consumers with the intention of maintaining flow during the interview.

Section C: Breeders

3. What are the main stakeholders involved in the broiler & layer breeder farming?
    - Are they small breeding businesses in Pakistan?

4. Could you please give an estimation on the market shares?

5. Could you please explain the main key differences between these companies?
    - Difference in where they get their birds?

6. Could explain to whom they sell their products?
    - The breeder hens and cocks?
    - Spent birds?

Section D: Hatcheries

7. What are the main stakeholders involved in the hatcheries?

8. Could you please give an estimation of the market shares?

9. Could you please explain the main key differences between these hatcheries companies?
    - What are their differences in the management of birds?

10. Could you please explain the main key differences between these companies?
    - Difference in where they get their birds?

11. Could explain to whom they sell their DOCs?
    - How these are sold to farmers?

Section E: Poultry producers

12. What are the main stakeholders involved in the poultry farms?

13. Could you please give an estimation of the market shares?

14. Could you explain the different types of poultry (broiler and layers) producers?
    - Could you explain if they have different geographical distribution?

15. Could you please give us an indication of the proportion
    - Of *farmers* in Pakistan belonging to the different production systems?
    - Of *poultry* in Pakistan produced by the different production systems?

16. Could you indicate their main differences in terms of:
    - Source of birds? How do they get these birds?

Looking at the flow diagram, for each type of production systems:

17. Could you please indicate what are the main flows associated with the selling of?
    A) Finished broiler birds
    B) Eggs
    C) Spent hens
    D) Manure
    E) Heads and shanks

18. Could you explain how different traders operate in the industry?

19. Are there other major stakeholders involved in the chain not mentioned yet?

20. Are there major groups dominating trade or retailing operations?
    - If so what are their market shares?

Section F: Poultry feed

21. What are the main stakeholders involved in the poultry feed production and distribution?

22. Could you please give an estimation on the market shares?

23. How do they sell their feed to farmers?

Section G: Animal Health and avian influenza

24. What are the main types of animal health service providers to poultry farmers?
    - How are they used by different production systems?

25. What are the main institutions and stakeholders involved in poultry disease/avian influenza detection and control?

26. What is the role of each institution/organization in controlling poultry disease/avian influenza?

27. How are poultry disease/avian influenza outbreaks reported by the different productions systems?

28. Are there any datasets available on the impact of avian influenza on farms?

Conclusion of the interview and thanking the interviewee for their time and help.
