# Supplementary material for: A Value Chain Approach to Characterize the Chicken Sub-sector in Pakistan
Source: Front Vet Sci. 2020 Jul 3;7:361. doi: 10.3389/fvets.2020.00361 (PMC7351015; doi:10.3389/fvets.2020.00361)
Supplement: Supplementary file 2 [file Data_Sheet_2.pdf]

Supplementary material 2

Questionnaire for key informant interviews and focus group discussions with the field veterinary officers and chicken farmers/traders to assess chicken industry structure and trade patterns

Royal Veterinary College

Before starting:

Introduction
Description of project and purpose of the interview
Time for questions
Presentations of consent form and acquisition of informant's signatures.

Questions

Section A: Understanding poultry industry structure

For my project, I want to identify the different types of poultry productions systems in Pakistan. This will help me to establish the type of farms that I could look at for assessing impact of avian influenza and cost-effectiveness of its vaccination.

1. In your view, what are the different broiler and layer production systems existing in your district/ area?
   - How would you classify them?
2. Could you explain if they have different geographical distribution?
3. Could you please give us an indication of the proportion:
   - Of *farmers* in your area belonging to the different production systems?
   - Of *poultry* in your area produced by the different production systems?
4. What is the level of integration of the production systems mentioned above?
5. Are there any associations or groups of poultry farmers in your area?
   - If so what is their role?

Section B: Questions to identify value chains

[Using the flipchart, the facilitator will use the next questions to create a flow diagram in consensus with participants showing the sources and destination of poultry or poultry products to/from the different systems identified in the previous section.]

6. What are the major inputs for your business (e.g. broiler and layer farming)?
7. What are the main stakeholders involved in the flow of inputs in your business?
   - Could you please explain how farmers get day old chicks?
   - What are the main stakeholders involved in the poultry feed production and distribution?
   - How do the farmers get feed on their farm?
   - Are there major groups dominating access to these inputs?
8. In your view, what are major outputs from your business (e.g. from broiler/layer farming)?
9. Could you please indicate what are the main flows associated for the selling of?
   - Prompt about:
     - Spent layers
     - Eggs
     - Live finished birds
     - Carcasses
     - Dead birds
     - Manure
     - Head and shanks
     - Offal
   - Prompts about destination of outputs:
     - Restaurants
     - Live bird/wet market
     - Caterers
     - Slaughter house
     - Supermarkets
     - Chicken stalls
   - Could you estimate the proportion of these outputs distributed to the different stakeholders?

[Facilitator to draw the different chains under different production systems in the flipchart.]

10. Could you explain how do the different traders operate in the industry?

Section C: Animal Health and avian influenza

11. What are the main type of animal health service providers available to the poultry farmers?
    - How are they used by different production systems?
12. What are the main institutions and stakeholders involved in poultry disease/avian influenza detection and control?
13. How are the poultry disease/ avian influenza outbreaks reported by the different productions systems?
14. Is there any centralised disease reporting centre/system for poultry diseases/avian influenza?
    - What are their responsibilities?
15. How do these organisations/institutions influence the farmers for controlling avian influenza?
16. How frequent have farmers experienced avian influenza outbreaks on their farms in your area?
17. How do poultry farmers manage their diseased or dead birds?
18. What are the major challenges in controlling avian influenza?
19. Are there any data sets available on the losses caused by the avian influenza on farms level?

Conclusion of the interview and thanking the participants for their time and help.
